# Meningioma Treated With Hypofractionated Stereotactic Radiotherapy Using CyberKnife®: First in the United Arab Emirates

**DOI:** 10.7759/cureus.21821

**Published:** 2022-02-01

**Authors:** Nandan M Shanbhag, Christos Antypas, Abdul K Msaddi, Sinead Catherine Murphy, Teekendra T Singh

**Affiliations:** 1 Radiation Oncology, Neuro Spinal Hospital, Dubai, ARE; 2 Medical Physics, Neuro Spinal Hospital, Dubai, ARE; 3 Neurological Surgery, Neuro Spinal Hospital, Dubai, ARE; 4 Oncology/Radiation Therapist, Neuro Spinal Hospital, Dubai, ARE

**Keywords:** space-occupying lesion, robotic stereotactic radiotherapy, hypofractionated stereotactic radiotherapy, meningioma, stereotactic radiotherapy, cyberknife

## Abstract

A 26-year-old premenopausal lady was referred to the Department of Oncology with headaches and easy fatiguability. She had presented with the same complaints a few years ago. At that time, imaging revealed a right falcine space-occupying lesion (SOL), for which she underwent an unsuccessful attempt of excision. Imaging studies confirmed that the SOL was progressive and arose from the meninges. Previous excision failure was due to a network of blood vessels around the tumor and critical structures such as the thalamus and the brainstem, which made any approach challenging.

The patient did not want further surgery and requested a non-surgical intervention. Considering the above, the case was discussed at the Multi-Disciplinary Tumor Board, and treatment with hypofractionated stereotactic radiotherapy using CyberKnife® was agreed upon. The patient received a total of 21 Gy in three fractions over six days and completed the treatment without any adverse reactions.

This is the first case treated with hypofractionated stereotactic radiotherapy using the CyberKnife® in the United Arab Emirates, which is an effective and safe modality to treat similar challenging cases.

## Introduction

Cancer is one of the leading causes of mortality and morbidity in the United Arab Emirates, with 4,807 new cases registered in 2020. Among the new cases, 2,155 were males, and 2,652 were females [1]. With advancements in medical science, we can apply novel treatment modalities to treat patients with fewer adverse reactions [2]. One such advancement is the robotic linear accelerator system, CyberKnife®, which can treat benign, malignant, and functional conditions with sub-millimeter accuracy [3].

While surgery continues to be the primary modality in many cases and can be recommended to a few patients, CyberKnife® can be used as a primary modality or adjuvantly after surgery to establish safe and effective treatment in challenging cases. In various circumstances, CyberKnife® can treat patients who are not candidates for surgery, and for whom delivering treatment would have been impossible. CyberKnife® is a non-invasive treatment for benign and malignant conditions where radiation therapy is indicated. The current case was reviewed during the multidisciplinary meeting (Tumor Board) attended by specialty physicians. The risks and benefits of the various treatments were discussed, and everyone agreed to the treatment plan involving hypofractionated stereotactic radiosurgery with CyberKnife®.

The indication for CyberKnife® was threefold; first, the proximity of the tumor to the critical structures; second, the midline and central placement of the tumor making it impossible to be excised in toto; and third, the patient refused to undergo any further surgical intervention after the failed first attempt fearing long-term morbidity as sequelae of subsequent attempts at excision. The patient discussed the treatment plan, including the adverse effects and other alternative treatment options, and the treatment was delivered safely after obtaining written informed consent.

## Case presentation

A 26-year-old premenopausal lady initially visited the hospital for neurosurgical assessment with complaints of easy fatigability and dizziness with occasional headaches in the morning. She was diagnosed with polycystic ovarian syndrome in 2017 when she developed hormonal imbalance disorder. She also complained of on-and-off low back pain, and the investigation revealed no obvious pathology. In 2019, when the severity of headaches became more intense, the patient underwent imaging procedures, which revealed a space-occupying lesion (SOL) in the brain. She underwent craniotomy outside of the United Arab Emirates, and tumor resection was attempted but was unsuccessful. In March 2021, repeat magnetic resonance imaging (MRI) with contrast was requested to assess the tumor. MRI showed a falcine mass, with homogenous enhancement, with a major diameter of less than 3 cm on either axis. Physical assessment revealed that the patient was alert and oriented, had no motor and sensory deficit, and all her cranial nerves and reflex functions were intact. The performance status was Eastern Cooperative Oncology Group Performance grade 1, which suggested that the patient could not perform physically strenuous activities but was ambulatory and able to carry out light or sedentary work [4]. She did not have any neurological deficits other than weakness in the left arm.

Investigations

**Figure 1:**
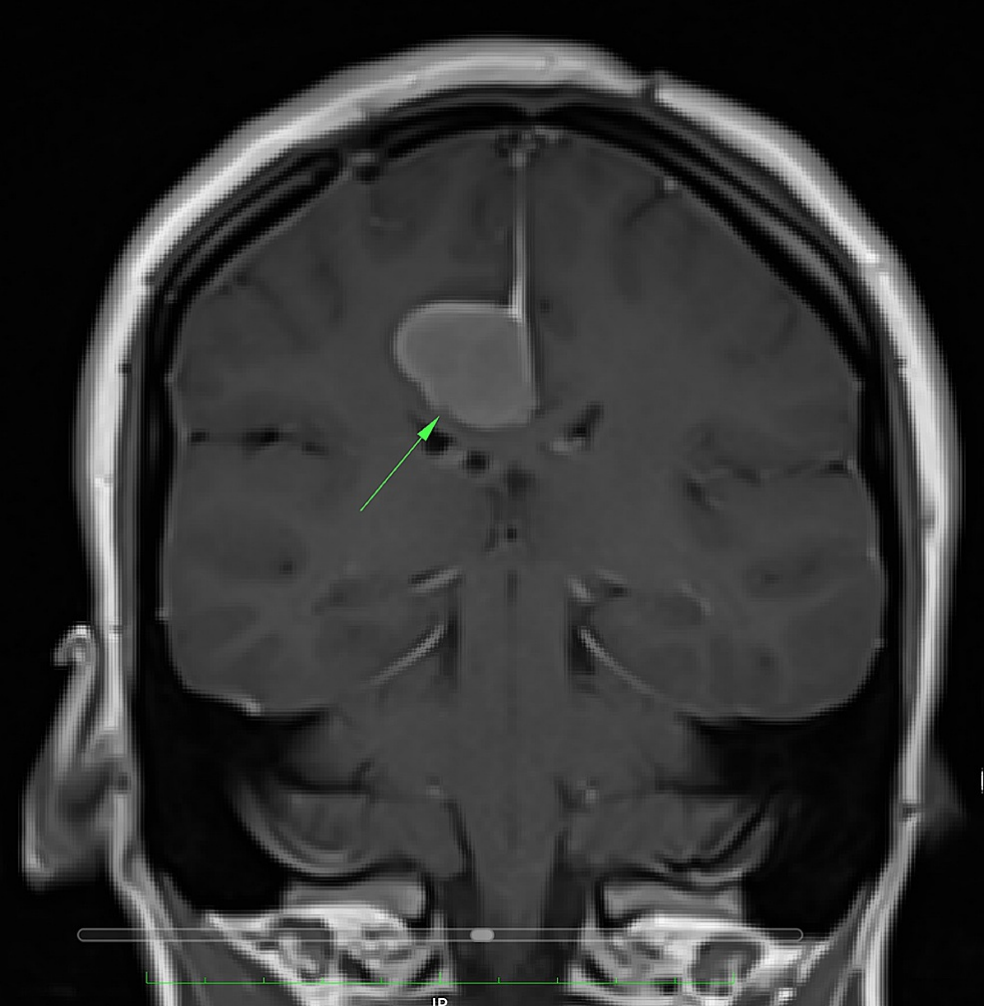
Coronal T1 MRI with contrast showing the space-occupying lesion marked by the arrow. MRI: magnetic resonance imaging

**Figure 2:**
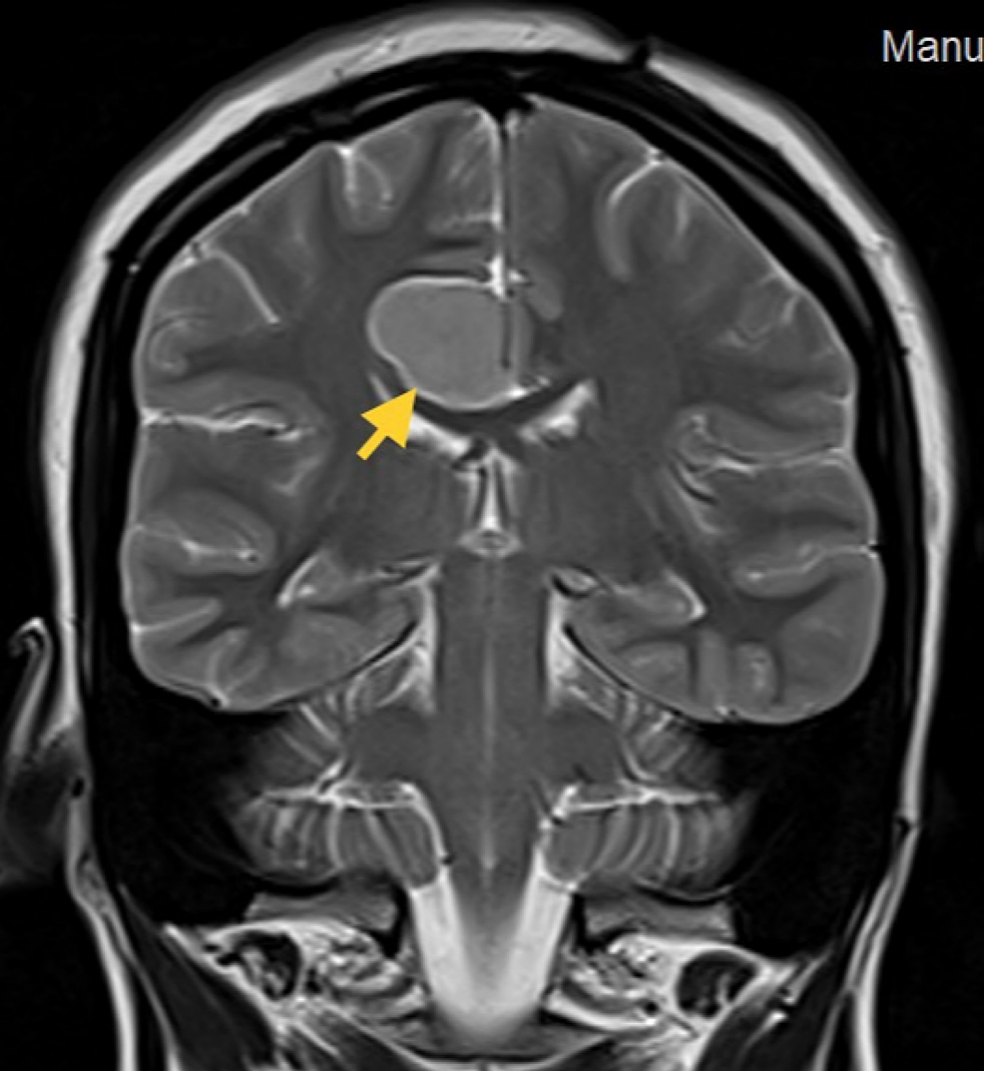
Coronal View T2 MRI with contrast showing the space-occupying lesion marked with the yellow arrow. MRI: magnetic resonance imaging

An MRI of the brain with contrast was done on December 08, 2021, which identified a focal ovoid extra-axial lesion in relation to the right-sided surface of the falx cerebri over the right low frontoparietal area near the inferior aspect of the right hemisphere. It measured 27 × 20 mm in cross-section and extended for a vertical height of approximately 21 mm. The lesion was smooth, irregular, and well-defined, showing a low to isointense signal in T1 (Figure 1), a bright signal in the T2 (Figure 2), and diffusion restriction in the diffusion map of the brain. The lesion was compressing the gray matter of the underlying brain with no abnormal signal in the underlying brain parenchyma, including compression and deformity of the posterior body of the corpus callosum reaching the splenium. In the postcontrast series, dense homogenous enhancement of the lesion could be seen which became very well illustrated with a dense enhancement, along with the enhancement of the adjacent meningeal lining confirming its meningeal origin.

Differential diagnosis

The patient presented with a unique challenge in that surgery was attempted but abandoned because the neurosurgeon feared excessive hemorrhage and damage leading to long-term morbidity given the proximity to critical structures. The patient refused second surgery and preferred non-interventional techniques. The characteristics of the mass in the imaging studies were most likely that of a meningioma, although a low-grade glioma was also observed in the same location.

Treatment

The treatment included simulation, contouring, plan generation, plan quality assurance, plan approval, and treatment delivery. During simulation, the patient was immobilized with a customized, non-invasive 2.4 mm thermoplastic mask formed over the face and affixed to an acrylic baseplate frame attached directly to the CyberKnife® couch. For patient comfort, a custom-formed pillow (AccuFormTM, Civco Medical Systems, Orange City, FL, USA) was used and shaped around the base of the head to hold its shape indefinitely. No external fiducial markers were used because of the capacity of the image guidance system in CyberKnife® during set-up and treatment. A non-contrast computed tomography scan of the head and a contrast-enhanced T1-weighted MRI, both with 1 mm slice thickness, were acquired and fused for delineating the target and the organs at risk.

**Figure 3:**
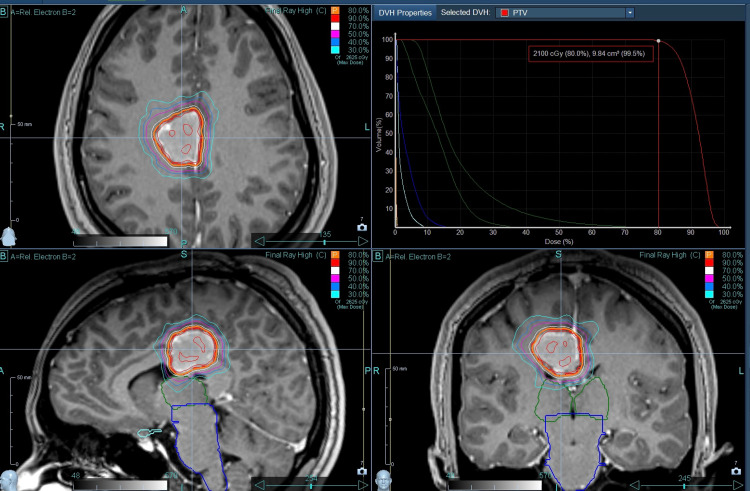
Accuray® CyberKnife® Precision® plan with the isodose distribution and the dose-volume histogram.

Plan evaluation was done with close observation of the isodose distribution and the dose-volume histogram and was approved after the set parameters were met (Figure 3).

For treatment delivery with image guidance, the patient was positioned on the treatment Robo-couch in the same position as during simulation using in-room lasers. The six-dimensional Skull Tracking mode was used. For initial patient alignment, orthogonal kilovoltage X-ray pairs were acquired using an in-room imaging system and compared with the planning digitally reconstructed radiographs using the two-dimensional-three-dimensional image registration method [5]. Initial set-up errors were detected and corrected by automated Robo-couch movement, thus bringing the patient to the treatment position. After the treatment started, real-time deviations were continuously recorded and adjusted by the robotic arm. The robot can automatically correct up to ±10 mm in all translational axes and up to ±1.5 degrees in all rotational axes without moving the patient [6,7]. Imaging intervals varied between 20 seconds and 150 seconds depending on the patient’s movement, minimizing intrafraction inaccuracy.

Outcome and follow-up

The patient completed the treatment without any complications, and on the one-month follow-up after treatment was doing very well.

## Discussion

Meningiomas are the most frequent primary brain tumors, accounting for nearly 30% of all nervous system tumors [8]. Although the majority of meningiomas are indolent and benign, the tumor’s location near critical structures can lead to significant morbidity and mortality. The incidence of meningioma increases with age, with a higher incidence in females. The predominance in females can be explained by hormone exposure, with nearly 10% of meningiomas expressing estrogen and progesterone receptors [9-11]. The World Health Organization has classified meningiomas as Grade I, II, and III. The recurrence is the highest with Grade III tumors, amounting to approximately 50-90% [12].

Most often, tumors are asymptomatic and discovered on incidental neuroimaging or at the time of autopsy [13-15]. If symptoms are present, usually due to mass effect on critical structures, patients present with visual disturbance, hearing loss, higher mental function deficiency, and weakness in extremities. Seizures are the most common presentation of parasagittal and falcine meningiomas [16]. The size and symptoms of meningiomas are critical deciding factors for managing these tumors. For small and asymptomatic meningiomas, close follow-up with neuroimaging and clinical examination is sufficient. For large or symptomatic meningiomas, the primary modality of treatment is surgery, with gross total resection being the goal. If gross total resection is not feasible, maximal safe total resection followed by adjuvant radiotherapy is recommended.

Radiation therapy as a primary modality is acceptable for inoperable and symptomatic meningiomas. Stereotactic radiosurgery has emerged as a feasible treatment modality, both as a primary treatment and in an adjuvant setting [17,18]. The advantage of CyberKnife® stereotactic radiosurgery is that it is non-invasive and precise in radiation delivery due to the interfraction tracking with image guidance. On long-term follow-ups, several series have reported 10-year local tumor control ranging 80-100% [19-22]. The critiques of these results have been that tumor control varies widely and depends primarily on tumor factors such as location, size, and duration of follow-up. A common observation is that meningiomas in the parasagittal and non-basal areas are prone to a higher risk of normal tissue complications, with peritumoral edema being the most common [23-26]. Some studies have observed a 40% risk of developing complications, including peritumoral edema [27-29]. Hypofractionated stereotactic radiosurgery/radiotherapy has been used successfully to mitigate the risk to a certain extent [30,31]. While stereotactic radiosurgery delivers a high dose of radiation in a single session, hypofractionated regimens deliver substantially high doses divided into multiple fractions of up to five fractions. This achieves a good balance between maximum tumor control and acceptable or lower normal tissue complications [32].

## Conclusions

Inoperable brain tumors pose a significant challenge, more for young patients where resection can lead to significant morbidity. In recent times, radiation has emerged as a champion modality providing treatment for complicated cases. When combined with precision planning and treatment delivery with sub-millimeter accuracy, tools such as CyberKnife® offer patients a new lease of life.
